# Genetics and Breeding for Glandless Upland Cotton With Improved Yield Potential and Disease Resistance: A Review

**DOI:** 10.3389/fpls.2021.753426

**Published:** 2021-10-06

**Authors:** Jinfa Zhang, Tom Wedegaertner

**Affiliations:** ^1^Department of Plant and Environmental Sciences, New Mexico State University, Las Cruces, NM, United States; ^2^Cotton Incorporated, Cary, NC, United States

**Keywords:** cotton, glandless cotton, genetics, breeding, pest responses

## Abstract

Glandless cotton (devoid of toxic gossypol) can be grown as a triple-purpose crop for fiber, feeds, and food (as an oil and protein source). However, its sensitivity to insect pests and its low yield due to the lack of breeding activities has prevented the realization of its potential in commercial seed production and utilization. Since the mid-1990s, the commercialization of bollworm and budworm resistant Bt cotton and the eradication of boll weevils and pink bollworms have provided an opportunity to revitalize glandless cotton production in the United States. The objectives of this study were to review the current status of genetics and breeding for glandless cotton, with a focus on the progress in breeding for glandless Upland cotton in New Mexico, United States. Because there existed a 10–20% yield gap between the best existing glandless germplasm and commercial Upland cultivars, the breeding of glandless Upland cultivars with improved yield and disease resistance was initiated at the New Mexico State University more than a decade ago. As a result, three glandless Upland cultivars, i.e., long-staple Acala 1517-18 GLS, medium staple NuMex COT 15 GLS, and NuMex COT 17 GLS with Fusarium wilt race 4 resistance were released. However, to compete with the current commercial glanded cotton, more breeding efforts are urgently needed to introduce different glandless traits (natural mutations, transgenic or genome-editing) into elite cotton backgrounds with high yields and desirable fiber quality.

## Background

Cotton (*Gossypium* spp.) is the most important fiber crop in the world and one of the most important oilseed crops along with soybean, rapeseed, sunflower, and peanut.^1^ Currently, cottonseed, traditionally treated as a by-product in cotton production, is primarily used for cooking oil and animal feed (Cherry et al., 1978, 1981; Cherry, 1983; Cherry and Leffler, 1984), and provides about 14–19% of the farm-gate value in cotton production.^2^ Cotton produces 150 kg of cottonseed for every 100 kg of lint fibers produced (O’Brien et al., 2005). Cottonseed oil is predominantly used in food service due to its exceptional high-temperature frying characteristics and is also used in processed foods where it contributes to extended shelf life. Unlike other plant seed oils, the toxic gossypol in cottonseeds needs to be chemically removed during seed crushing and oil refining before it can be used for human consumption. The cottonseed crushing industry estimated this to add 20% of the cost in cottonseed processing. Glandless cotton will produce cottonseeds devoid of gossypol which will be much easier and cheaper to convert to refined edible oil. Glandless cotton will also make cottonseeds an important protein source for human consumption especially in major cotton-growing developing countries (Rathore et al., 2020).

The growth of the global human population demands an increased production of food, fiber, and feeds. However, crop production is restrained by climate conditions, including the increased drought frequency and salinity in agricultural lands. Because of the reduction in insect pressure owed to the widespread use of the insect-resistant transgenic Bt cotton, there is a recent renewed interest in the research and usage of glandless Upland cotton (*G. hirsutum* L.). Glandless cotton can produce glandless seeds which may be used for human food (as an oil and/or protein source) and animal feed, even though it was reported that glandless cottonseed products might cause hypersensitive allergic reactions in a very small percentage of humans (Atkins et al., 1988). Potentially, the commercial production and processing of glandless cottonseeds can significantly increase the net income for both cotton producers and processors, making cotton a triple-purpose (fiber, feed, and food) crop.

The objectives of this study were to review the current status of genetics and breeding for glandless cotton, with a focus on the progress in breeding for glandless Upland cotton in New Mexico, United States (Zhang et al., 2014a,b,c,d, 2016a, 2017, 2018, 2019b 2019c; Zhang et al., 2020b). Other relevant studies on glandless cotton from New Mexico, such as responses to insects and crop management practices, were also mentioned in this study. A review of the processing and utilization of glandless cottonseed can be found in the study by Lusas and Jividen (1987). Recently, Rathore et al. (2020) published a comprehensive review on glandless cotton research including the biosynthetic pathway of gossypol and related terpenoids, animal feeding studies, and human nutrition studies.

## Sources and Genetics of the Glandless Trait

There are four genetic sources for the potential glandless cottonseed production. The first natural glandless mutant (with no gland on the plant and seed) in Upland cotton was discovered by McMichael (1959), which is conditioned by two recessive alleles, namely, *gl*_2_ and *gl*_3_ (McMichael, 1960). The two glandless genes, *gl*_2_ and *gl*_3_, did not exhibit any deleterious effects on the lint yield and fiber quality, however, contributed to the lower cottonseed yield, smaller seed size, and higher lint percentage in glandless cotton (Halloin et al., 1978). The two genes were later mapped to chromosomes (*Gl*_2_ to A12 and *Gl*_3_ to D12) through linkage analysis (Lee, 1965; Endrizi et al., 1985; Kohel, 1979) and molecular mapping (Dong et al., 2007). While normal glanded cotton carries homozygous dominant *Gl*_2_*Gl*_2_*Gl*_3_*Gl*_3_ (provides ca. 1% of the gossypol content in seeds and plants), the distribution of glands or gossypol content may be changed by the other alleles of the two genes and other glanded loci (*Gl*_1_, *Gl*_4_, *Gl*_5_, and *Gl*_6_) with different alleles from different genetic sources (Wilson and Smith, 1977; Lee, 1978; Endrizi et al., 1985; Calhoun, 1997).

The second glandless cottonseed source was an induced dominant glandless mutation, identified in “Bahtim 110,” which was derived from the irradiation of seeds from the Egyptian cotton (*G. barbadense* L.), “Giza 45” (Afifi et al., 1966). The glandless (with no glands in the plants and seeds) trait is conditioned by a dominant gene, *Gl_2_^*e*^*, which is allelic to *gl*_2_, although marginal or reduced glandedness in the cotyledons and hypocotyls were also observed in heterozygotes (Kohel and Lee, 1984). *Gl*_2_^*e*^ is epistatic to *Gl*_3_ in that it suppresses the expression of the glanded *Gl*_3_ gene that renders glandless plants and seeds in the *Gl*_2_^*e*^*Gl*_2_^*e*^*Gl*_3_*Gl*_3_ genotype. The genetic basis was later confirmed in other genetic backgrounds or advanced backcrosses (De Carvalho and De Macedo Vieira, 2000; Hinze and Kohel, 2006). For example, when this gene was incorporated in the commercial Brazilian Upland cultivar CNPA Precoce 2, a few glands were shown in the cotyledons of the heterozygous plants, indicating the incomplete dominance of the glandless trait, but the F_1_ seed had gossypol-free content, similar to the homozygous *Gl_2_^*e*^Gl_2_^*e*^* parent (De Carvalho and De Macedo Vieira, 2000). The *Gl_2_^*e*^* gene was fine mapped within a 15 kb region in chromosome A12 with only one gene (with 1428 bp) encoding for a basic helix–loop–helix (bHLH) transcription factor of 476-amino acids (Cheng et al., 2016; Ma et al., 2016), which was named as the *Gossypium* pigment gland formation gene (*GoPGF*) by Ma et al. (2016). Through homologous gene cloning and sequencing, Ma et al. (2016) further showed that the single “T” nucleotide insertion between 735 and 736 bp in the coding region of *GhPGF_A12^*m*^* is the likely causal gene for the recessive glandless *gl*_2_ allele in A12, while the single “A” nucleotide insertion between 916 and 917 bp in the coding region of *GhPGF_D12^*m*^* is likely the causal gene for the recessive glandless *gl*_3_ allele in D12. The two single nucleotide insertions caused the premature translation termination of the encoded transcription factors, making the truncated proteins non-functional.

The third source, a glanded plant and glandless cottonseed trait with delayed pigment gland morphogenesis, was found in a few wild diploid Australian cotton species, as represented by *G. bickii*. Attempts have been made for the interspecific crossing between these Australian species and Upland cottons, such as through a tri-specific hybridization between amphidiploid [(*G. arboreum* × *G. bickii*) F_1_ (2n = 52, A_2_A_2_G_1_G_1_), (*G. herbaceum* × *G. astrale*) F_1_ (2n = 52, A_1_A_1_G_2_G_2_), or (*G. thurberi* × *G. sturtianum*)F_1_ (2n = 52, D_1_D_1_C_1_C_1_)] and *G. hirsutum* (Bi et al., 1999; Zhu et al., 2004, 2005; Liu et al., 2015). There have also been attempts through synthetic hexaploid avenues such as between the *G. hirsutum*–*G. bickii* amphidiploid (2n = 78, AADDG_1_G_1_) and *G. hirsutum* (Tang et al., 2018), or between the *G. hirsutum*–*G. raimondii* amphidiploid (2n = 78, AADDD_5_D_5_) and *G. sturtianum* (Bi et al., 1999). However, the trait has not been successfully transferred into Upland cotton for utilization in research due to the difficulty in interspecific introgression. Therefore, the genetic basis (such as the number of genes, gene effects, and chromosomal locations) for the characteristic- glanded plant and glandless cottonseed trait with delayed pigment gland morphogenesis is still unknown.

The fourth source, a genetically engineered (GE) Upland cotton, producing ultra-low gossypol cottonseeds (ULGCS), was produced by Sunilkumar et al. (2006), based on RNA interface (RNAi) to silence the delta-cadinene synthase gene(s) driven by a seed-specific α-globulin promoter. The ULGCS and glanded plant trait were reported to be stable under both greenhouse and field conditions (Rathore et al., 2012; Palle et al., 2013). As compared with the Coker 312 used for the transformation, the three ULGCS lines tested appeared to have similar leaf terpenoid and gossypol content, lint yield, seed protein content, and fiber quality, but 4–8% higher seed oil content (Rathore et al., 2012; Palle et al., 2013). As compared with the normal glanded Coker 312, the developing cotyledons of the transformed ULCGS cotton appeared to have a lower level of gossypol content, incurring a higher level of damage from African cotton leafworms, *Spodoptera littoralis* (Boisd.), while the fully expanded true leaves showed similar responses to the insect (Hagenbucher et al., 2019). Rathore et al. (2020) have recently provided a detailed account of events that led to the development of ULCGS. The petition for the deregulation of the GE low-gossypol trait was recently approved by both the United States Department of Agriculture (USDA) (in Oct. 2018) and the Food and Drug Administration (FDA) (in Sept. 2019) (Rathore et al., 2020). The introduction of this trait to elite cotton has been ongoing but has made slow progress due to the concerns about international regulatory hurdles. There were other attempts in developing transgenic or non-transgenic glandless cotton. For example, most recently, Li et al. (2021) reported using the temperature-sensitive CRISPR/LbCpf1 (LbCas12a) mediated- genome editing system to successfully create non-transgenic gossypol-free Upland cotton. Other genes (Tian et al., 2018; Janga et al., 2019; Gao et al., 2020), involved in gland formation and the gossypol biosynthetic pathway, can be targets for genetic modification to produce glandless cottonseed.

## Breeding for Glandless Upland Cotton

Since the discovery of the double recessive glandless (*gl*_2_*gl*_2_*gl*_3_*gl*_3_) cotton by McMichael (1959, 1960), numerous efforts have been made in breeding and researching glandless cotton in the United States until the 1970s, which were summarized in a conference entitled “Glandless cotton-its significance, status and prospects” published in 1978 (Anonymous, 1978). Numerous improved glandless germplasm lines were developed and released. Thaxton et al. (1987) reported progress in developing glandless cotton in the multi-adversity resistant (MAR) program and showed that the new glandless GCANH-1-83 MAR strain had improved yield potential, fiber quality, and disease resistance that was equal to the latest releases of Tamcot cultivars such as the CAMD-E but was slightly less resistant to insects (Thaxton et al., 1998). Seed companies and public breeders developed several commercial glandless cultivars; however, glandless cotton was grown in only a very limited acreage in central Texas and the Texas High Plains in the mid-1980s. However, dehulled, roasted, and whole glandless kernel products and associated business did not gain much of the market, due to quality control, insects, and marketing issues, in addition to possible allergenic reactions (Hinze and Kohel, 2012). Since then, there were intermittent breeding activities until the late 1990s (Shepherd, 1982; Smith and Niles, 1988; Owen and Gannaway, 1995; Dobbs and Oakley, 2000). Using the glandless trait as a genetic marker, breeding populations using Upland cotton lines with varying gland densities involving glandless cotton were recently developed (Gutiérrez et al., 2006; Scheffler and Romano, 2012; Hinze et al., 2014). The potential of glandless cottonseeds in cotton production and product development as a triple-purpose crop (fiber, feeds, and food) has not been realized in the United States.

In the 1970s, glandless germplasm (with the *gl*_2_*gl*_2_*gl*_3_*gl*_3_ genotype) was introduced from the US to other cotton-growing areas including Africa and China, and the breeding for glandless Upland cotton was subsequently conducted. Hau (1987) reported that 24,000 hectares were cultivated in Ivory Coast in 1984 with the glandless variety, ISA BC2, created by IRCT-IDESSA in Bouak. China released at least 20 glandless Upland cotton cultivars (Ma et al., 2016).

Although the genetic study on *Gl*_2_^*e*^ was conducted in the US (Kohel and Lee, 1984), no breeding activities were recorded using this glandless source. The National Cotton Germplasm Collection does not have accessions possessing the *Gl*_2_^*e*^ gene, and the glandless accessions are all homozygous recessive *gl*_2_gl_2_*gl*_3_*gl*_3_. However, the dominant glandless *Gl*_2_^*e*^ gene was introduced from *G. barbadense* into Upland cotton through interspecific hybridizations and backcrossings in China (Yuan et al., 2000). Based on a field study on seven pairs of near-isogenic lines (NILs) with glanded or dominant glandless traits in Upland cotton, Yuan et al. (2000) did not detect significant associations between the dominant glandless gene and most agronomic, fiber, and seed quality traits; however, the oil or protein content was higher in one glandless line than in its glanded NIL. Advanced backcross populations involving the *Gl_2_^*e*^* gene were used in a quantitative trait locus (QTL) mapping for yield, yield component traits, fiber quality, and disease resistance by the Yuan group (Shi et al., 2016, 2019; Li et al., 2019). A new Upland cotton cultivar with glanded plants and seeds with low gossypol content was reportedly released (Zhang et al., 2001); however, its authenticity was not independently verified.

## Responses to Insect Pests in Glandless Cotton

Glands containing toxic gossypol and other terpenoid aldehydes are distributed in most of the tissues and organs of cotton, which plays an important role in defending cotton against insect pests that feed on its tissues and organs. Glandless cotton eliminates this protection and incurs heavier insect damage than conventional glanded cotton (Benedict et al., 1977). In addition, glandless cotton is susceptible to field mice and foraging by livestock and wildlife animals. This has become one of the most important limiting factors preventing the commercial production of glandless cotton. Therefore, a more detailed review of the responses of glandless cotton to insect pests is warranted here.

### Boll Weevils (*Anthonomus grandis* Boheman)

The boll weevil had been a major cotton insect in the US Cotton Belt until its successful eradication in the early 2000s. Stephens and Lee (1961) compared the feeding and oviposition preferences of boll weevils among a standard Upland cotton cultivar and three mutant strains, namely, hairy, hairy-glandless, and hairy-glandless-red, in laboratory tests and a field test, and no discriminations were observed between glandless and glanded cotton. Through a 2-year field study to compare two pairs of glanded and glandless NILs in large plots and 13 pairs of glanded and glandless NILs in small plots in Mississippi, Jenkins et al. (1967) showed that glandless cotton possessing the two glandless genes, *gl*_2_ and *gl*_3_, were not more susceptible, although the boll weevils were slightly larger on some glandless NILs. In another companion laboratory study, Maxwell et al. (1966) showed that the weevils fed significantly less on six glandless lines than on their glanded parental NILs and laid significantly more eggs on three glandless lines than on their glanded NILs, while there was no difference detected in the oviposition rate between the nine pairs of glandless and glanded NILs. The weevils reared on square powder diets made from three glandless lines were larger than those with similar diets made from their glanded NILs, while the opposite was true for the other paired lines. No significant difference was observed in the developmental period of weevils between the glandless and glanded paired NILs studied. The results from both studies led to the conclusion that the double recessive glandless trait will not increase boll weevil susceptibility in some genetic backgrounds. In a 4-year field study at Stoneville, Mississippi, Merkl and Meyer (1963) showed that the level of the punctured squares by weevils on the glandless cotton was the same as the cotton lines with smooth leaves or the nectariless trait, and factors such as plant height, leaf color, and growth characteristics affected the percentage of the infested squares. Through an inheritance study in the F_1_, F_2_, and backcross progeny of the cross with Upland Deltapine Smooth Leaf (DPSL), Buford et al. (1968) showed that among the 252 cotton lines tested, “S. I. Seaberry” (*G. barbadense*) produced the lowest oviposition rate by the weevils, and possessed a genetic factor (X factor) that suppresses weevil oviposition. However, the gossypol content in cotton is related to the preference of weevils in feeding and oviposition. When the gossypol contents increased to levels higher than normal in cotton, the weevils preferred feeding and oviposition on glandless (with no gossypol) or normal-glanded (normal gossypol content) strains (Singh and Weaver, 1972).

### *Helicoverpa* spp.

Glandless cotton is highly susceptible to Helicoverpa (often called heliothis), including tobacco budworm [*H. virescens* (F.)], corn earworm [*H. zea* (Boddie)] and bollworm (*H. armigera* Hubner), a serious pest in cotton in most cotton-growing countries. It appears that Helicoverpa cannot metabolize the gossypol but excretes it to a certain degree (Montandon et al., 1987). The first stage larvae of *H.* spp. were shown to avoid feeding on the gossypol glands of anthers or other parts of the cotton plant due to the presence of allelochemicals including anthocyanins, but then non-selectively consumed the glands (Belcher et al., 1983; Parrott et al., 1983; Parrott, 1990), and they were, therefore, feeding less frequently and resting more on the glanded cotton than on glandless cotton (Schmidt et al., 1988). Consequently, the *H. zea* or *H. virescens* larvae feeding on glandless cotton grew faster and gained more weight (Lukefahr et al., 1966; Meredith et al., 1979; Montandon et al., 1986, 1987; Scheffler et al., 2012), had increased survival rates and shorter developmental time, and caused more damage on the cotton (Mullins and Pieters, 1982; Zummo et al., 1983; Wang et al., 2008; Pierce et al., 2012, 2014; Garnett et al., 2013). The number of dominant glanded alleles is associated with the preference of *H. virescens* in that the larvae favored glandless (*gl*_2_*gl*_2_*gl*_3_*gl*_3_) seedlings, and the preference was decreased as the number of *Gl*_2_ and *Gl*_3_^*ral*^ alleles increased (Wilson and Lee, 1971). Wilson and Lee (1971) indicated that the number of pigment glands on the cotyledonary petiole and percentage of seed gossypol were correlated with seedling damage and the number of larvae that *H. virescens* left on the seedlings. Glandless cotton not only lacks gossypol but also lacks or contains small quantities of volatile terpenes (Elzen et al., 1985). Lukefahr and Houghtaling (1969) found that cotton with high gossypol (HG) contents (1.7%) inhibited the growth of *H.* spp. Shaver et al. (1980) observed a significant linear relationship between the reduction in larval weight of tobacco budworms and gossypol content in squares. Commercial Upland cotton normally exhibits an approximate 3 (+)-gossypol to 2 (−)-gossypol ratio and Stipanovic et al. (2006) showed that both (+)-gossypol and (−)-gossypol were equally inhibitory to *H. zea* larvae, although (+)-gossypol is less toxic than (−)-gossypol to non-ruminant animals. In a feeding study to compare three pairs of glanded and glandless NILs using leaves and artificial diets with five levels of gossypol content, Kong et al. (2010) found that the glanded cotton and diets with higher levels of gossypol decreased larval weights and moth eclosion rates and delayed the development of the larvae and pupae of *H. armigera*; and the larvae that fed on the glanded cotton leaves were more tolerant to two insecticides (cyhalothrin and monocrotophos). In studies comparing 14 pairs of normally glandless and glanded cotton NILs, the increased larval growth of *H. zea* was only observed when they fed on diets of glandless cotton (Lukefahr et al., 1966; Oliver et al., 1971), but there were no significant differences between any glandless vs. glanded pairs in the number of eggs oviposited or in the number of squares and bolls damaged by the *H. zea* in field plots (Oliver et al., 1970a, b). Additionally, in field studies, Jenkins et al. (1966) showed that *H. zea* caused similar damages on the Rex Smooth leaf glanded and glandless cotton lines and glanded Deltapine Smooth Leaf, but the glandless Acala incurred higher damage than glanded Acala cotton.

The resistance of glandless cotton to the damages from *H.* spp. can be improved to the level of glanded cotton by using Bt genes. Benedict et al. (1993) showed that two glandless lines with Bt genes reduced the larval survival of *H. zea* to nearly zero with no damage to the squares and bolls, as compared to the average of 24–33% survival rates on glanded non-Bt NILs. However, it appeared that no commercial cotton cultivars possessing both glandless and Bt traits were developed and commercialized.

### Armyworm (*Spodoptera* spp.)

In field conditions, Bottger et al. (1964) observed that beet armyworms [*S. exigua* (Hübner)] preferred glandless cotton over glanded cotton. In laboratory bioassays, McAuslane and Alborn (1998) confirmed that *S. exigua* larvae strongly preferred feeding on glandless cotton when given a choice between glanded and glandless cotton plants. In a greenhouse study, McAuslane and Alborn (2000) further showed that neonate beet armyworms avoided feeding on the gossypol-rich young leaves of glanded cotton plants because they moved down the plant to feed on the older leaves when placed on the terminal foliage; however, the larvae feeding on glandless plants were evenly distributed within the plant. In a no-choice laboratory study, even though the larvae feeding on young or mature leaves from the glanded or glandless plants had similar survival rates, the pupae and adults from the larvae reared on the young or old leaves of glanded cotton weighed significantly less than those on the glandless plants. In addition, the time to pupation and adult emergence was significantly longer for the larvae fed on glanded plants. In New Mexico, Pierce et al. (2012, 2014) and Garnett et al. (2013) reported that beet armyworms caused higher leaf damage and took a shorter time to pupation with 2–6 times higher survival rates when fed on glandless Acala GLS as compared to glanded Acala 1517-99. In Israel, Meisner et al. (1977c) compared the effects of different cotton strains with different gossypol contents of leaves on the development of *S. littoralis* larvae and showed that the larvae fed on HG (1.23%) strain had lower weight, required longer time for pupation, and reduced pupal weight and pupation rate. The *S. littoralis* larvae fed on only half of the food containing an extract from the HG cotton strain than that containing an extract of a glandless strain (Meisner et al., 1977a). Zur et al. (1978) determined the gossypol content of the cotyledons and true leaves during the growing season in 12 HG Upland cotton lines, a normal glanded cultivar, and a glandless line, and the *S. littoralis* larvae feeding on the glandless cotyledons gained the highest weight and the lowest on the three HG lines. Zur et al. (1980) further confirmed the value of HG cotton strains in suppressing *S. littoralis* and *Earias insulana* (Boisd.) populations as compared with a glandless strain and a normal glanded Upland cultivar, in unsprayed fields of two production regions. Similar to *S. exigua*, an avoidance strategy was reported for the *S. littoralis* larvae that avoided gossypol-rich young leaves by migrating from the young leaves to the older leaves (Anderson et al., 2001). Meisner et al. (1977b) showed that glandless cotton reduced the effectiveness of phosfolan (2-(diethoxyphosphinylimino)-1,3-dithiolane in controlling *S. littoralis*.

### Plant Bugs (*Lygus* spp.)

Plant bugs are a serious mid-season insect pest problem in cotton production, as they prefer to feed on the squares and young bolls. The western tarnished plant bug (WTPB) (*L. hesperus* Knight) feeding on glandless cotton increased its growth rate and survival of nymphs by twofold, resulting in a 2.5 times greater WTPB population and a 57% reduction in cotton bolls in California (Tingey et al., 1975; Benedict et al., 1981; Leigh et al., 1985). However, the susceptibility of glandless cotton is dependent on genetic backgrounds. In a field cage study, Leigh et al. (1985) showed that 32 glandless lines supported 1.9- to 2.5-fold higher WTPB than the glanded “Acala SJ-2,” whereas 20 other glandless lines did not differ from Acala SJ-2 in WTPB populations. Thirty-seven glandless lines were selected to further evaluate the effect of cotton genotypes on nymphal survival, growth rate, and adult oviposition preference in a greenhouse. The results indicated that glandless cotton that is no more susceptible to WTPB than the glanded cultivar, Acala SJ-2, could be developed, indicating that other genetic factors can reduce the susceptibility of glandless cotton. Based on a 2-year multi-location study in the San Joaquin Valley (SJV), California, Goodell et al. (2001) found little difference among the Acala, Upland, and Pima cultivars for arthropod affinity; however, the glandless Acala cultivar C-166 had a significantly higher total population of WTPB than the other glanded cultivars in three locations over the 2 years. In Mississippi, Meredith et al. (1979) showed that glandless cotton had a higher yield loss upon infestation from tarnished plant bugs (TPB) [*L. lineolaris* (Palisot de Beauvois)] than without TPB; and among 99 glandless lines, the least sensitive glandless lines possessed combinations of nectarilessness, hirsuteness, or rapid fruiting ability, characteristics which were previously found to be less susceptible to TPB.

### Thrips (*Frankliniella* spp. and *Thrips tabaci* Lind.)

Thrips are important insect pests at the seedling stage. Based on a leaf area reduction to measure the resistance of thrips (Rummel and Quisenberry, 1979), Quisenberry and Rummel (1979) showed that morphological traits such as glandlessness (*gl*_2_*gl*_3_) did not provide the plant with resistance to thrips, except for Pilose (*H*_2_) which was highly resistant. In China, glandless cotton was also more susceptible to thrips (Fang et al., 1995). Based on a field study of 11 glanded Pakistan Upland cotton and a glandless check (Rizwan et al., 2021), the glandless check had significantly higher populations of whiteflies [*Bemisia tabaci* (Genn.)], thrips, and jassids (*Amrasca devastans* Dist.), and the leaf gland density was significantly and negatively correlated with the populations of the three insect pests. However, in an extensive field study in New Mexico (Zhang et al., 2014b, d), many glandless cotton lines were compared with the glanded control Acala 1517-08 and other glanded lines for their resistance to the Western flower thrips [*F. occidentalis* (Pergande)]. These lines were divided into four replicated field tests, each with 32 genotypes. In the same field, many glanded commercial cultivars and breeding lines were divided into three other tests to compare with the glanded Acala 1517-08 and Acala 1517-99. Overall, the glandless cotton had similar or lower damages from thrips than the glanded cotton, indicating that the glandless trait may serve as a genetic factor for suppressing damage from thrips. As compared with Acala 1517-08 which represented one of the most thrips resistant genotypes among the glanded cotton tested, glandless Acala GLS and many glandless selections were more resistant, indicating that unknown genetic factors other than the glandless trait also affect thrips resistance in cotton (Zhang et al., 2014b). The results were corroborated by the development of many thrips resistant lines in an Acala 1517-08 × Acala GLS cross. Similar results were obtained in a greenhouse study (Larson et al., 2015; Larson, 2019).

### Other Insects

Many secondary insects or insects that did not use normal glanded cotton as a major host were found to infest glandless cotton and cause significant damages. In Arizona, Bottger et al. (1964) observed that, in addition to *H. zea* and *S. exigua*, black fleahoppers [*Spanogonicus albofasciatus* (Reuter)], grape colaspis beetles [*Maecolaspis flarida* (Say)], cutworms (undetermined species), pill bugs (*Porcellio* spp.), and rodents also preferred eating glandless cotton before attacking glanded cotton under field conditions. Maxwell et al. (1965) observed greater susceptibility in several glandless experimental lines than in their glanded NILs to cotton leafworms [*Alabama argillacea* (Hüibner)], bollworms, grape colaspis beetles, and *Gastrophysa cyanea* (Melch) in Mississippi; and Japanese beetles (*Popilla japonica* Newman) damaged the leaves of glandless lines extensively in North Carolina. Also in Mississippi, Jenkins et al. (1966) observed a preference in feeding and oviposition on all glandless lines from adult insects such as *M. flavida*, *G. cyanea*, and *A. argillacea* that usually did not cause damage to glanded cotton, causing considerable damage to all glandless lines. Glandless cotton was also more sensitive to two-spotted spider mites [*Tetranychus urticae (*Koch)] (Schuster et al., 1972; Bailey and Meredith, 1983). After the glandless cotton germplasm was introduced and tested in other countries or regions such as China, India, Pakistan, Brazil, and Africa, higher insect pest pressures were also found on glandless cotton than on glanded cotton. For example, glandless cotton lines were heavily infested by sucking pests throughout the growing season in India, including cotton leafhoppers (*Amrasca biguttula biguttula* Ishida), jassids, *B. tabaci*, and *T. tabaci* (Bhatnagar and Sharma, 1991).

### Predators

Benedict et al. (1977) reported that more predators were collected in glandless plots compared to glanded plots in California. In New Mexico, Ellington et al. (1984) showed that glandless cotton supported larger phytophagous populations than glanded cotton, but the HG genotypes did not affect the phytophagous populations and its effect on the population of predators was ambiguous. Also in New Mexico, Pierce et al. (2015, 2016, 2017) showed overall similarity in predation rates between glanded and glandless cotton based on multi-year field studies. In Brazil, Silva et al. (2002) did not observe any difference in the population density of predators between glandless and glanded genotypes.

### Controversial Results

Montandon et al. (1986) fed glanded and glandless cotyledons to *A. argillacea*, a specialist on Gossypieae, and showed that the *A. argillacea* survived equally well on either cotton type but had significantly higher larval weights by feeding on the glanded leaves. The results suggested that glanded cotton may not lessen but even increase the impact of the adapted specialists on cotton. It was reported in China that glandless cultivars were more resistant to aphids (*Aphis gossypii* Glover) and spider mites damage than glanded ones (Fang et al., 1995). In Arizona, a glandless Pima mutant (*G. barbadense*) was found to suffer significantly less seed damage from pink bollworms [*Pectinophora gossypiella* (Saund.)] than the glanded check Pima S-4 or S-5 (Wilson et al., 1977, 1979). In Pakistan, among the 20 cotton genotypes evaluated for their resistance or tolerance to *A. devastans*, *Scirtothrips dorsalis* (Hood), *T. tabaci*, *B. tabaci*, *E. insulana* (Boisd.), *E. vittella* (F.), and pink bollworms (Baloch et al., 1982), the glandless, nectariless and gossypol-free genotypes were susceptible to the attacks by *A. devastans*, while the glandless, nectariless, glabrous, hairy and okra-leaf genotypes were more susceptible than the others to *E.* spp. and pink bollworms. In Brazil, no differences were observed between the glandless and glanded genotype population density for cotton aphids, *T. tabaci*, cotton leaf worms, and pink bollworms (Silva et al., 2002). It should be noted that because most of these studies with controversial results came from germplasm lines with different morphological traits, different genetic backgrounds, experimental designs, growing stages, and environmental conditions may affect the effect of glandless cotton on the growth and development of different insect pests. Because genotype × trait interactions often exist, NILs in different genetic backgrounds should be developed and used to compare glandless and glanded cotton.

McCarty et al. (1983) studied Upland cotton lines with different morphological traits in multiple locations without early season insect control, and the results showed that the nectariless lines had high adaptability, while the lines with other morphological traits, including glandlessness, did not, due likely to the varying insect pressures in different locations. In New Mexico and the other areas of the US Cotton Belt such as Arizona and Far-west Texas, the overall insect pest pressure has been low, due to the successful eradication of boll weevils and pink bollworms and the reduced damage of budworms/bollworms by growing transgenic Bt cotton. Thus, glandless cotton may not suffer from heavy insect damages and high yield losses as before. The responses of glandless cotton to phytophagous populations and predators should be studied under the current production conditions.

## Responses to Disease Infections in Glandless Cotton

It is well known that gossypol and other related terpenoid aldehydes (TA) are phytoalexins (Rathore et al., 2020). Studies have shown that gossypol and other TA were induced in the roots or leaves of glanded cotton upon being infected by different pathogens such as soil-borne fungi (*Verticillium dahliae* Kleb.) causing Verticillium wilt. *Fusarium oxysporum* f. sp. *vasinfectum* (G.F. Atkinson) Snyder & H.N. Hansen (FOV) causing Fusarium wilt, and *Xanthomonas citri* pv. *malvacearum* causing bacterial blight; and high or elevated TA contents may be related to the resistance to these diseases (for a review, refer to Rathore et al., 2020). However, it is known that most glanded cotton lines are susceptible to these pathogens, while there are resistant glandless cotton genotypes. Khoshkhoo et al. (1994) compared the concentrations of TA, including gossypol, in the roots and leaves between several susceptible and resistant glanded and glandless cotton lines to root-knot nematodes (RKN) [*Meloidogyne incognita* (Kofoild and White) Chitwood]. They found that the TA content and its increase in the root were not associated with RKN resistance. It is now understood that the two glanded genes (on A12 and D12) and the two major resistance genes/QTLs (one on A11 and another on D02) for RKN resistance are not linked. Similarly, there is no direct linkage between glanded genes and QTLs when it comes to the resistance to bacterial blights (Zhang et al., 2020a), Verticillium wilt, and Fusarium wilt (Abdelraheem et al., 2017). In several fields and greenhouse studies, we have also found that glandless cotton was not more susceptible to Verticillium wilt than glanded cotton (Larson et al., 2015; Larson, 2019). Several new glandless cotton lines were shown to be more resistant to leaf spots [*Alternaria alternata* (Fr.) Keissl.] in several field tests (Zhu et al., 2017, 2018). In addition, two new glandless Upland cultivars, namely, NuMex COT 15 GLS and NuMex COT 17 GLS, were found to be as resistant to Fusarium wilt caused by *Fusarium oxysporum* f. sp. *vasinfectum* (G.F. Atkinson) Snyder & H.N. Hansen (FOV) race 4 as the resistant glanded Pima cotton PHY 802 RF and PHY 811 RF (Zhang et al., 2020b). However, we are presently unsure if the resistance is related to a gene closely linked to the dominant glandless gene (*Gl_2_^*e*^*), chromosome A12, or derived from their FOV race 7-resistant Chinese Upland parent. Overall, there is no direct genetic relationship between glanded or glandless genes and the resistance to diseases or tolerance to abiotic stresses in cotton. However, under natural infections in the field, glandless cotton appeared to be more susceptible to Southwestern cotton rust (*Puccinia cacabata* Arth. and Holw.) than glanded cotton (Zhang et al., 2017).

## Responses to Herbicides in Glandless Cotton

Foster et al. (1994) compared the prometryn tolerance between glanded and glandless isolines in a growth chamber and the field and showed that glandless (*g1_2_gl_3_*) cotton incurred higher photosynthetic inhibition and longer durations of inhibition by the herbicide, with 20–56% higher visual leaf injury ratings and 44–66% lower yield than the corresponding glanded isoline. The results demonstrated that lysigeneous glands enhance prometryn tolerance in cotton. Furthermore, the *G1_2_G1_2_g1_3_g1_3_* had lesser leaf injury than the *g1_2_g1_2_G1_3_G1_3_* isoline, indicating that the prometryn tolerance of *Gl*_2_ is higher than *Gl*_3_. Zhang et al. (2019b) conducted a field study with four replicated tests to evaluate 81 cotton genotypes, including 8 Pima and 73 Upland genotypes, and their responses to halosulfuron (Sandea) at the 4–5th true-leaf stage. Three glandless cotton lines were significantly more sensitive than all the glanded cotton tested except for one sensitive glanded cultivar. However, in another study (Zhang et al., 2021), results indicated that glandless cotton was not more sensitive to trifloxysulfuron (Envoke) than glanded cotton when treated at the 7-true leaf stage.

## Breeding for Commercial Glandless Cotton Cultivars in New Mexico

### The Yield Gap Between Glandless Cotton and Commercial Upland Cultivars

To understand the breeding progress in long-staple Acala cotton, Acala germplasm and the cultivars released in New Mexico and California since the 1930s were collected from the National Cotton Germplasm Collection and were evaluated in the early 2000s in a field in New Mexico (Zhang et al., 2019a). The yield of the glandless Acala was much lower than that of the glanded cultivars including the control Acala 1517-99 (Cantrell et al., 2000). We subsequently initiated breeding activities to develop glandless Acala cotton with improved lint yield and fiber quality. Zhang et al. (2014e) further stated the following: “in 2010, an Acala glandless cotton (Acala GLS) released in California in 1999 (Dobbs and Oakley, 2000) was introduced and tested together with Acala 1517-99 and Acala 1517-08 (Zhang et al., 2011) in a national cotton variety test in Las Cruces, NM.” To further enrich the glandless germplasm collection and evaluate their yield potential, obsolete and exotic glandless germplasm were collected and observed in a field in 2011–2012. Because of the noticeable phenotypic variation including segregation in the glandless trait, single plants were selected for seed increase and progeny tests. Existing glandless cotton germplasm was evaluated in eight replicated tests in Las Cruces, NM, the United States in 2010–2013, for lint yield, fiber quality, and their adaptability to the New Mexico cotton production conditions where Acala cotton has been traditionally grown. Zhang et al. (2014a, c) showed that the glandless Acala GLS produced only 65–80% lint yield of Acala 1517-08 and 46–75% lint yield of transgenic cultivars in multiple tests. Idowu et al. (2011, 2012, 2013a, 2014) also showed that Acala GLS produced 50% less lint than the glanded Acala 1517-99 in New Mexico. Even though the two other glandless cotton lines (JACO developed in Louisiana and STV GL developed in Mississippi) yielded 12–21% more than Acala GLS, they only yielded 57–63% of Acala 1517-08 and 51–55% of the commercial transgenic PHY 375 WRF. In another replicated test in 2012, 14 obsolete US glandless lines were tested and most of them yielded below 80% of the lint of Acala 1517-08.

### Selection Within Existing Glandless Germplasm

The original glandless germplasm lines and their selections were advanced to several replicated field tests in Las Cruces, NM, United States (Zhang et al., 2014a). Among the selections, most lines produced lint yields less than 70% of the Acala 1517–08, and three selections within three lines (Acala G8160, SA 2455, and Acala GLS) brought the yield up to 82–89% of Acala 1517-08. Therefore, there existed a significant yield gap (i.e., ca. 10–20%) between the best glandless germplasm and commercial cotton. Furthermore, significant differences in fiber quality were found between selections within the same glandless germplasm lines, indicating genetic variation in fiber quality traits. The results indicated that residual genetic variation still existed, although most of the glandless germplasm lines were developed through pedigree selections from different cross combinations.

Meredith and Bridge (1982) estimated that the US national mean genetic gain in cotton yield improvement was 0.74% per year. For the New Mexico Cotton Breeding Program, the cotton yield gain due to breeding was 1.4% per year between 1930 and 2004 (Zhang et al., 2005, 2019a). Thus, it would take 7–13 years of breeding efforts to fill this 10–20% yield gap, i.e., to bring the yield up to the current level of glanded commercial cultivars. This is a difficult task because genetic improvement in lint yield still will be made for glanded commercial cotton by seed companies.

### Crossbreeding for Glandless Upland Cotton

In 2010–2011, cross-breeding for glandless Upland cotton was initiated by crossing Acala 1517 with obsolete glandless cotton. In 2012, 35 new glandless lines were tested in a replicated trial in Las Cruces, NM. Approximately 70 exotic glandless lines were collected and grown in the field with selections made. In 2013, 150 new glandless breeding lines were evaluated in several replicated field tests. In 2014, tests on new glandless lines were performed at three locations (Las Cruces, Artesia, and Tucumcari) in New Mexico and 14 locations across the Cotton Belt. In the greenhouse, the 150 new glandless lines tested in the field in 2013 were evaluated for thrips and Verticillium wilt resistance. Under both the greenhouse and field conditions, 30 glandless lines plus two glanded checks were also tested for Verticillium wilt resistance. In 2015, several new glandless lines were tested at 14 locations across the Cotton Belt and also in three locations in New Mexico (Las Cruces, Artesia, and Tucumcari). Two replicated field tests were further performed in both Las Cruces and Artesia with each test having 30 glandless lines and two glanded checks for field performance and Verticillium wilt resistance.

From 2016 to present, increased breeding activities for glandless cotton have continued, which included: (1) 400–600 progeny rows; (2) several replicated field tests with 32 lines each; and field and/or greenhouse tests for resistance to thrips, Verticillium wilt, FOV race 4, bacterial blights, cotton rust, and leaf spots on an annual basis. It should be pointed out that, all the field trials in New Mexico did not receive any insecticide applications including seed treatments since 2010, when pink bollworms, bollworms, and plant bugs did not cause significant lint yield losses.

Due to the 10–20% yield gap between the best high-yielding glandless cotton and current commercial cultivars, crossbreeding should be taken into consideration to significantly increase the yield potential of glandless cotton so its potential as a triple-purpose crop can be fully realized. In the New Mexico Cotton Breeding Program, the glanded Acala 1517-08 cotton was first used to cross with the glandless Acala GLS, followed by repeated pedigree selections (Zhang et al., 2016a). Eighteen glandless individuals were first selected from 500 F_2_ plants and tested in F_3_ progeny rows, which were used as a base population for further single plant selections, followed by a progeny test. In the end, 77 F_6_ lines were selected for further replicated field testing. Five lines produced 90–96% of the Acala 1517-08 lint yield. There was no positive transgressive segregation for lint yield, lint percentage, and boll weight in this Acala/Acala cross because negative transgressive segregations occurred frequently for the three traits and fiber strength. Negative transgressive segregations occurred more frequently although positive transgressive segregation was observed for lint yield and micronaire.

The above Acala/Acala cross resulted in the development of the long-staple glandless Upland cultivar Acala 1517-18 GLS from an F_4__:__6_ line, which carries the double recessive glandless genes *gl*_2_*gl*_2_*gl*_3_*gl*_3_ (Zhang et al., 2019c). In the cultivar release notice, Zhang et al. (2019c) summarized its field performance, as following: “This new glandless cultivar was tested in 11 replicated field trials in New Mexico in 2013–2016 and 14 tests across 11 US states in 2015. Acala 1517-18 GLS produced 93% of the lint yield in Acala 1517-08 across all the tests without observed seed-cotton losses from rodents.” But it yielded 30% more lint than Acala GLS. Acala 1517-18 GLS had a similar fiber quality with Acala 1517-08 and Acala GLS in fiber length, uniformity, strength, and micronaire, but had a similar or higher elongation and similar or lower short fiber content. In addition to the higher seed index, Acala 1517-18 GLS had longer and stronger fibers, higher fiber length uniformity and elongation, but lower micronaire and short fiber content than most of the other medium-staple commercial checks. As compared to Acala 1517-08, it was more resistant to Alternaria leaf spots and had a similar or higher level of resistance to Verticillium wilt. Acala 1517-18 GLS represent the first contemporary long-staple (with fiber length > 30 mm) Acala cotton cultivar with the glandless trait.

### Introgression Breeding for Glandless Upland Cotton

Since the rediscovery of the Mendelian Laws in genetics in 1900, interspecific introgression genetics and breeding between Upland and Pima cotton (*G. barbadense*) have been extensively conducted by numerous scientists (Zhang et al., 2014e). However, only a few, if any, commercial cultivars were developed from this approach, due to the hybrid breakdown between the two closely related cultivated tetraploid cotton species. The hybrid breakdown is the reduced hybrid viability and/or fertility segregating in F_2_ and later generations, due to the complementary effects of numerous recessive genes from the two parental species. Under field conditions, almost no plants from an interspecific cross exhibited similar productivity with both its parents. In a genetic sense, the hybrid breakdown is transgressive segregation in a negative direction. Therefore, a breeder should first mitigate the hybrid breakdown caused by the negative transgressive segregation. Since 1985, J. Zhang has been working on introgression genetics and breeding in cotton (Zhang, 2011; Zhang et al., 2014e). As a result of the long-term effort, two glandless Upland cultivars, namely, NuMex COT 15 GLS and NuMex COT 17 GLS with FOV race 4 resistance, were developed and released from the advanced backcrossing between Upland and dominant glandless *G. barbadense* (Zhang et al., 2016b, 2020b). The release of the two NuMex COT cultivars represented the first successful attempt in introducing incomplete dominant glandless allele *Gl_2_^*e*^* from *G. barbadense* to Upland cotton and developing commercial cultivars with acceptable yield and fiber quality characteristics in the US. The Brownfield Seed & Delinting Company (BS&D) in west Texas listed Acala 1517-18 GLS and NuMex COT 17 GLS (listed as Acala 1117) for seed sale.^3^ The two NuMex COT glandless cultivars were the result of a long-term effort of introgression breeding in transferring desirable genes and traits from Pima to Upland cotton.

Both NuMex COT 15 GLS and NuMex COT 17 GLS have the same pedigree, “derived from an advanced backcross progeny of a cross between the *Gl_2_^*e*^* allele donor “Bahtim 110” (*G. barbadense)* and glanded Upland cotton CRI 12, followed by five backcrosses with a glanded Upland CRI 35 as the recurrent parent” (Zhang et al., 2016b, 2020b). In the advanced backcross population, they were selected as two glandless progenies out of a mixture of glandless and glanded plants. When evaluated in two naturally infected fields in California, the two cultivars were resistant to FOV race 4, with resistance levels similar to the resistant checks- Pima PHY 802 RF and PHY 811 RF.^4^ However, they were more resistant than Acala 1517-08, when evaluated in a greenhouse in New Mexico. The two cultivars have been used in crossbreeding to developing new germplasm lines to build resistance to FOV race 4 (Ulloa et al., 2020). The two cultivars were tested in 4–7 replicated field trials in New Mexico in 2013–2017 and 11 tests across 9 US states in 2014. Both cultivars yielded more lint than Acala GLS, and NuMex COT 17 GLS yielded more than NuMex COT 15 GLS as it produced 16 and 13% more lint yields than Acala 1517-18 GLS, and NuMex COT 15 GLS, respectively, and reached 93% of the yield of the glanded Acala 1517-08. Both were classified as having medium to long staples with a fiber quality similar to other commercial medium staple cultivars, but inferior to both Acala cultivars. NuMex COT 15 GLS had longer and stronger fibers than NuMex COT 17 GLS, but the latter had a higher lint percentage and was specially adapted to the Mississippi Delta because it was the top yielder in the region. The two glandless cultivars responded to thrips and Verticillium wilt similarly to Acala 1517-08 but were less susceptible to Alternaria leaf spots. NuMex COT 17 GLS was resistant to four races including race 18 of bacterial blights (see text footnote 4). The high yield potential of NuMex COT 17 GLS, together with other glandless lines, was evaluated in different soil types and under different crop management practices such as planting date, nitrogen rate, potassium application, deficit irrigation, and reduced tillage in New Mexico (Idowu et al., 2013b, 2015, 2016, 2018; Sultana et al., 2018).

## Perspective

Glandless cotton (conditioned by two recessive genes, *gl*_2_*gl*_3_) had received great attention in the US between the 1960s and the 1970s after its first discovery by McMichael (1959). During this period, most of the public breeding and genetic programs were involved in developing glandless Upland cotton lines or cultivars, resulting in the development of numerous improved glandless lines and a few commercial cultivars. The two glandless genes were also transferred into Pima cotton through interspecific crossings and backcrossings. Because of the unusually high insect pressure associated with glandless cotton, many breeders and geneticists developed NILs with glanded and glandless traits through backcrossing to study the genetic efforts of the two glandless genes on yield, fiber, and seed quality in Upland cotton, and its responses to insects. There appears to be no direct genetic association between glandless genes and responses to diseases, nematodes, and abiotic stresses. Through collaborations between entomologists and cotton breeders, extensive field, greenhouse, and laboratory studies were conducted to compare the susceptibilities to insect pests between glandless and glanded cotton under similar genetic backgrounds. As compared with glandless cotton, larvae of boll weevils, bollworms, and armyworms have developed an avoidance mechanism to avoid glands or gossypol-rich young leaves while searching for food, and they, therefore, feed less and rest more on glanded cotton. Thus, these and other insect pests grow faster and gain more bodyweight with higher survival rates and shortened time to pupation as adults on glandless cotton. In some genetic backgrounds, no significant differences in the susceptibility to boll weevils and bollworms/budworms were observed between glandless and glanded NILs under field conditions, due likely to some unknown genetic resistance factors that could compensate or alleviate the susceptibility of glandless cotton. This led breeders to search for traits or genetic factors to be used to alleviate the susceptibility to insects in glandless cotton. For example, the plant height, leaf color, growth characteristics, and an X genetic factor in a Sea-Island accession (*G. barbadense*) were found to confer resistance to boll weevils in glandless cotton. Glandless cotton possessing combinations of nectarilessness, hirsuteness, or rapid fruiting ability were less susceptible to plant bugs. However, these traits could still not provide adequate protection against major insect pests in both glanded and glandless cotton. Equally importantly, the market for seed processing and utilization was not developed to demand commercially grown glandless cotton in the 1970 and 1980s.

However, it is time to revitalize the breeding, research, and utilization of glandless cotton in the US and the world. First of all, the current cotton production conditions are greatly different from 10 to 20 years ago. Boll weevils and pink bollworms are no longer a production issue in the US because of successful eradication programs. After more than 25 years of the commercial production of Bt cotton in the US and its widespread use in other cotton-producing countries, *Helicoverpa* spp. including budworms, earworms, bollworms, and other lepidopteran pests are under effective control. Plant bugs, aphids, and spider mites are not major pests in many cotton production regions; and usually, thrips do not need chemical controls because cotton seedlings can outgrow their damage. Second, there are more amendable genetic sources of glandless cotton such as the dominant glandless trait (*Gl_2_^*e*^*), transgenic cotton, producing ULGCS, and non-transgenic glandless cotton through genome editing, in addition to the double recessive glandless trait that was most extensively studied. However, due to the lack of breeding activities in developing commercially competitive glandless cotton, the large yield gap between the best glandless cotton and current commercial cultivars has prevented farmers from growing glandless cotton. Therefore, it is urgently imperative that more breeders and geneticists are engaged in the effort to use different sources of glandless traits and to develop elite and commercial high-yielding glandless cotton with good fiber quality and resistance to insects and diseases. Glandless traits should be introduced into commercial cultivars with insect-resistant Bt and herbicide-tolerant cotton.

## Author Contributions

JZ drafted the manuscript. TW edited and approved the final manuscript. Both authors contributed to the article and approved the submitted version.

## Conflict of Interest

The authors declare that the research was conducted in the absence of any commercial or financial relationships that could be construed as a potential conflict of interest. The handling editor declared a past co-authorship with one of the authors JZ.

## Publisher’s Note

All claims expressed in this article are solely those of the authors and do not necessarily represent those of their affiliated organizations, or those of the publisher, the editors and the reviewers. Any product that may be evaluated in this article, or claim that may be made by its manufacturer, is not guaranteed or endorsed by the publisher.

## Footnotes

1
www.statista.com/statistics/267271/worldwide-oilseed-production-since-2008

2
www.cotton.org/econ/cropinfo/costsreturns/usa.cfm

3
www.brownfieldseed.com

4
https://rbtn.cottoninc.com
